# Supplementary material for: Knowledge, practices and seroprevalence of Taenia species in smallholder farms in Gauteng, South Africa
Source: PLoS One. 2020 Dec 18;15(12):e0244055. doi: 10.1371/journal.pone.0244055 (PMC7748137; doi:10.1371/journal.pone.0244055)
Supplement: S1 Appendix — (PDF) [file pone.0244055.s001.pdf]

S1 Appendix: Consent form for farmers to participate in the study

Porcine cysticercosis: Seroprevalence and associated risk factors in selected areas in Gauteng

Introduction

This research study is being conducted by Nothando Shongwe at the University of Pretoria, South Africa, to investigate the Seroprevalence of porcine cysticercosis and its associated risk factors in selected areas in Gauteng.

Procedure

The participants will be asked to respond to closed and open-ended questions prepared on a. questionnaire. The interview will take approximately 30 minutes for individual consumers and for farmer groups the exchange will take approximately 45 minutes. The questions for the consumer questionnaire will include details about sanitary activities, meat preparation and knowledge on *Taenia solium* transmission. Questions on the farmer questionnaire will entail details about pig husbandry practices, sanitary practices and knowledge on *T. solium* transmission, prevention and control.

Risks

There are no risks for participation in this study.

Benefits

There will be no monetary benefits from participation in this study. The information that will be obtained during the study on the burden of porcine and possible human cysticercosis will aid in guiding the implementation of control strategies relating to *T. solium* infection and transmission in the community. In addition, the data from this study will be used for further research.

Participation

Participation of respondents in this study is voluntary. The participants may refuse to participate or withdraw at any time during the study. However, non-participation will disadvantage the community in that the results obtained from the study will not be a true representation of the prevalence in the study area. A misrepresentation of the results will affect the level of importance of *T. solium* control strategies in the study area.

Confidentiality

All the information will remain confidential. The data will be recorded as group data with no identifying information and data will be destroyed should participants withdraw from the study. The data obtained from the participants will be secured in Institution for a period of 15 years. Only the Supervisors, the researcher and other persons directly involved in the research will have access to the information.

Access to the researcher

If participants have enquiries or wish to withdraw from the study, they may contact Nothando Shongwe at (071) 926 3932

Location …………………………………………..

Date ……………………………………………….

Signature of Participant……………………………….

Signature of Researcher……………………………
