# Supplementary material for: Knowledge, practices and seroprevalence of Taenia species in smallholder farms in Gauteng, South Africa
Source: PLoS One. 2020 Dec 18;15(12):e0244055. doi: 10.1371/journal.pone.0244055 (PMC7748137; doi:10.1371/journal.pone.0244055)
Supplement: S2 Appendix — (PDF) [file pone.0244055.s002.pdf]

**Interviewer**			
**Code**	Date	City/Town	
		**DEMOGRAPHICS**	
**1**	Gender (Tick applicable answer)	☐ Male	☐ Female
**2**	Age group		
**3**	Education status		
		☐ Primary	
		☐ Secondary	
		☐ Tertiary	
		☐ None	
	Economic characteristics		
**4**	What is your source of income?		
	☐ Formal employment	☐ Farming	☐ None
		**FARMING PRACTICE**	
**5**	Are you a farmer?	Y ☐ N ☐	
**6**	What is your involvement in the farm?		
	☐ Self-employed	☐ Employed by pig owner	
**7**	How many years have you been farming with pigs?		
**8**	What kind of training did you receive in pig management?		
		☐Inhouse	☐Other farmers
		☐Veterinary extension	☐None
**9**	What is your purpose of farming with pigs? (select all applicable options)		
		☐ Selling live pigs	☐ Own consumption
		☐ Selling pork meat	
**10**	How would you classify your pig production system?		
		☐ Intensive (Confined)	
		☐ Semi-intensive	
		☐ Free-range	
**11**	How many pigs do you keep?		
**12**	What is the type of pig breed?		
		☐ Large white	☐ Duroc
		☐ Landrace	☐ Mixed
**13**	What is the source of the current pig stock on the farm?		
	☐ Born on farm	☐ Institution	
	☐ Auction	☐ Other farmers	
		☐ Other (specify)	
**14**	Do you introduce new pigs on the farm?		Y ☐ N ☐
**15**	If Yes, where do you source the stock?		

16	What is the type of feed provided to the pigs?	
	☐ Kitchen waste	☐ Combination of feed types
	☐ Commercial feed	☐ Other (specify)
	**TRANSMISSION**	
	**If answer to questions y/n is no, skip follow up explanation questions**	
17	Have you heard about cysticercosis?	
		Y ☐ N ☐
18	Have you received any education on public health awareness about pork measles?	
		Y ☐ N ☐ Y ☐ N ☐
19	Have you received any education on public health awareness about neurocysticercosis?	Y ☐ N ☐
20	If answer is yes, where did you acquire this information? (please specify)	
21	Do you know how a pig acquires cysticercosis?	Y ☐ N ☐
22	If yes, please specify	
23	Do you know where to find cysts in a pig?	Y ☐ N ☐
24	If Yes, please specify	
25	Have cysts ever been detected on your pigs?	Y ☐ N ☐
26	If yes, by who?	
	☐ Farmer	☐ Animal health Technician
	☐Veterinary doctor	☐Other
27	Do you know how the pig got infected?	Y ☐ N ☐
	If yes, please specify:	
28	How many animals were infected?	
29	What do you do to prevent and control the occurrence of pork measles in your enterprise?	
	**SANITATION**	
30	Do you have access to a toilet?	Y ☐ N ☐
31	If Yes, how often do you use the toilet?	☐ Always
		☐ Sometimes
32	If No, where do you defecate? (please specify)	

**33**	Do you use clean water and soap to wash your hands after defecating?		
		☐ Water only	☐ None
		☐ Both water and soap	
	**SLAUGHTER AND MEAT INSPECTION**		
**34**	Do you slaughter pigs at home?		Y ☐ N ☐
**35**	Do you inspect the meat?		Y ☐ N ☐
	**If answer is No, Skip to question 37**		
**36**	What do you look for when inspecting the meat? Explain:		
**37**	What do you do when abnormalities are found on the carcass?		
		☐ Discard	☐ Sell
		☐ Consume	☐ Other(specify)
	**HUSBANDRY PRACTICES**		
**38**	Do the pigs on your farm get dewormed?		Y ☐ N ☐
**39**	If Yes, what is the name of the dewormer used?		
	**IF NO, end of questions**		
**40**	Which age group of pigs are dewormed?		
		☐ All pigs	☐ Only piglets
		☐ Only adult pigs	
**41**	how often?		
		☐ Once a year	
		☐ More than once a year	
